# Whole genomes and transcriptomes reveal adaptation and domestication of pistachio

**DOI:** 10.1186/s13059-019-1686-3

**Published:** 2019-04-18

**Authors:** Lin Zeng, Xiao-Long Tu, He Dai, Feng-Ming Han, Bing-She Lu, Ming-Shan Wang, Hojjat Asadollahpour Nanaei, Ali Tajabadipour, Mehdi Mansouri, Xiao-Long Li, Li-Li Ji, David M. Irwin, Hong Zhou, Min Liu, Hong-Kun Zheng, Ali Esmailizadeh, Dong-Dong Wu

**Affiliations:** 1 State Key Laboratory of Genetic Resources and Evolution, Kunming Institute of Zoology, Chinese Academy of Sciences, Kunming, 650223 China; 2 Allwegene Technologies Inc., Beijing, 102209 China; 3 Biomarker Technologies Corporation, Beijing, China; 4 Department of Animal Science, Faculty of Agriculture, Shahid Bahonar University of Kerman, PB 76169-133, Kerman, Iran; 5 Kunming College of Life Science, University of Chinese Academy of Sciences, Kunming, 650204 China; 6 Department of Laboratory Medicine and Pathobiology, Banting and Best Diabetes Centre, University of Toronto, Toronto, ON M5S 1A8 Canada; 7 College of Landscape Architecture and Tourism, Agricultural University of Hebei, Baoding, 071000 China; 8 Pistachio Research Center, Horticultural Sciences Research Institute, Agricultural Research, Education and Extension Organization (AREEO), Rafsanjan, Iran; 9 Department of Agricultural Biotechnology, Faculty of Agriculture, Shahid Bahonar University of Kerman, Kerman, Iran; 10 Chinese Academy of Forestry Sciences, Beijing, China; 11 Center for Excellence in Animal Evolution and Genetics, Chinese Academy of Sciences, Kunming, 650223 China

**Keywords:** *Pistacia vera*, Crop domestication, Artificial selection, Genome

## Abstract

**Background:**

Pistachio (*Pistacia vera*), one of the most important commercial nut crops worldwide, is highly adaptable to abiotic stresses and is tolerant to drought and salt stresses.

**Results:**

Here, we provide a draft de novo genome of pistachio as well as large-scale genome resequencing. Comparative genomic analyses reveal stress adaptation of pistachio is likely attributable to the expanded cytochrome P450 and chitinase gene families. Particularly, a comparative transcriptomic analysis shows that the jasmonic acid (JA) biosynthetic pathway plays an important role in salt tolerance in pistachio. Moreover, we resequence 93 cultivars and 14 wild *P. vera* genomes and 35 closely related wild *Pistacia* genomes, to provide insights into population structure, genetic diversity, and domestication. We find that frequent genetic admixture occurred among the different wild *Pistacia* species. Comparative population genomic analyses reveal that pistachio was domesticated about 8000 years ago and suggest that key genes for domestication related to tree and seed size experienced artificial selection.

**Conclusions:**

Our study provides insight into genetic underpinning of local adaptation and domestication of pistachio. The *Pistacia* genome sequences should facilitate future studies to understand the genetic basis of agronomically and environmentally related traits of desert crops.

**Electronic supplementary material:**

The online version of this article (10.1186/s13059-019-1686-3) contains supplementary material, which is available to authorized users.

## Background

With reducing agricultural acreage and human population growth, feeding the world is becoming an increasing problem. Deserts take up about one third of the land surface area of Earth, are extreme environments that are barren landscapes where little precipitation occurs, and often have dry and alkaline soils, thus have hostile living conditions for most plant and animal life [1]. However, some crops can still be cultivated in some desert areas. Insight into the environmental adaptations and economic characters of these species should facilitate the planting and breeding of these crops in different desert regions, which might contribute to easing the world’s food crisis.

**Fig. 1:**
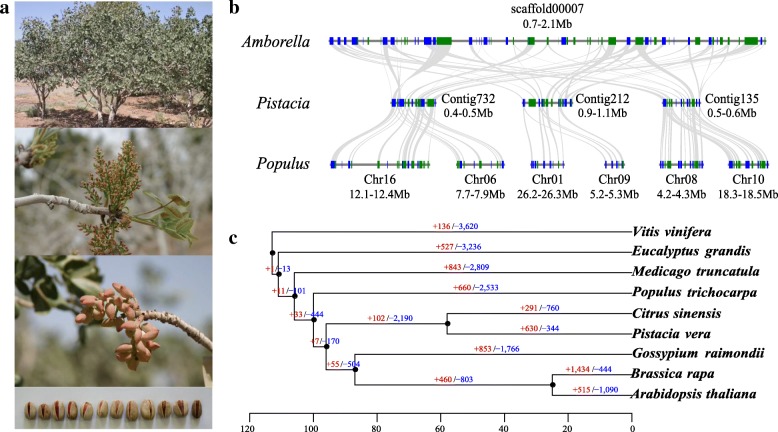
Pistachio genome evolution. **a** Tree, flower, immature seeds, and mature seeds of pistachio Ghazvini. **b** Example microsynteny analysis indicating that no lineage-specific whole genome duplication occurred in pistachio. Microcollinearity patterns between genomic regions from Amborella, pistachio and the populus. Rectangles represent predicted gene models, with blue and green showing relative gene orientations. Gray ribbons connect the matching gene pairs. **c** Expansion (red numbers) and contraction (blue numbers) of gene families in different plants

Pistachio (*P. vera*, 2*n* = 30, Fig. 1a) belongs to the Eudicots clade, Sapindales order, and Anacardiaceae family and is a member of the cashew family originating from Central Asia and the Middle East. It is a desert plant that is highly tolerant of saline soil. Pistachio nuts have recently become the fifth largest nut crop, with around 1024 kt harvested in 2015 (FAOSTAT. Food and Agriculture Organization of the United Nations Database, http://faostat.fao.org/). Iran and the USA were the major producers of pistachios, together accounting for 72.65% of the total world production in 2015, with the USA overtaking Iran in 2016 to become the country with the biggest pistachio production (FAOSTAT). In addition to its economic, nutritional, and medicinal values, pistachio is highly adaptable to abiotic stresses and is considered to be a species that tolerates drought and salt stresses, making it ideal for reforestation of arid and salinized zones [2].

Although the rapid development of genome sequencing has facilitated to discover genetic underpinning of many crop domestication and improvement, there are very few studies on pistachio. The genome size of pistachio has been estimated to be about 600 Mb with a high heterozygosity rate [3]. Moazzzam Jazi et al. [2] used a genome-wide transcriptome and discovered the salinity tolerance-related markers and stress response mechanisms by comparing two pistachio cultivars under control and salt treatment.

In the present study, to better understand the molecular evolutionary history underpinning pistachio domestication, we assembled a draft genome of pistachio and resequenced 107 whole genomes, including 93 domestic and 14 wild individuals of *P. vera* and 35 other genomes from different wild *Pistacia* species. Integrating genomic and transcriptomic analyses revealed expanded gene families (e.g., cytochrome P450 and chitinase) and the jasmonic acid (JA) biosynthetic pathway that are likely involved in stress adaptation. Comparative population genomic analyses revealed that pistachio was domesticated ~ 8000 years ago and that likely key genes for domestication are those involved in tree and seed size, which experienced artificial selection (Additional file 1). These genome sequences should facilitate future studies to understand the genetic underpinnings of agronomically and environmentally related traits of desert crops.

## Results and discussion

### Genome evolution of pistachio

We firstly sequenced the genome of the *P. vera* L cultivar by Illumina Hiseq 2500 platform from multiple paired-end libraries, including two small-insert libraries (270 bp and 500 bp) and six long-insert mate-pair libraries (3 kb, 4 kb, 8 kb, 10 kb, 15 kb, and 17 kb). A draft genome of 569.12 Mb was assembled, with contig and scaffold N50 sizes of 20.69 kb and 768.39 kb, respectively. (Additional file 2: Tables S1-S2, version1). To improve the continuity, we further generated a total of 4,038,150 filtered long reads with average lengths of 14,568 bp from 59 Gb sequencing data by Pacbio Sequel System. Finally, a draft genome of 671 Mb was assembled, with contig and scaffold N50 sizes of 75.7 kb and 949.2 kb, respectively (Additional file 2: Tables S2, version 2). The genome quality is compatible with the previously reported plant genomes (Additional file 2: Tables S2) and facilitates some convincing data analyses. The assembly size is a little larger than the estimated genome size, which is likely due to the high heterozygosity of pistachio (1.72%). The pattern has also been reported in other assembled genomes with high heterozygosity [4–6]. Transposable elements occupied 70.7% of the pistachio genome, of which 46.75% were long terminal repeat retrotransposons (Additional file 2: Table S3). A total of 31,784 protein-coding genes and 161 miRNAs were annotated by integrating different methods (Additional file 2: Tables S4-S5). Conserved Core Eukaryotic Gene Mapping Approach (CEGMA) analyses indicated that 96.94% of the core protein-coding genes were recovered in our assembled genome. The assembly sequence was assessed with BUSCO v3.0.2b which found 1361 complete gene models out of 1440 (94.51%) and 29 fragmented (2.01%); 18.96% of complete genes were found in more than one copy (Additional file 2: Table S6).

We first performed a comparative genomic investigation to assess the palaeohistory of this species. Phylogenomic analysis using genes extracted from single-copy families in nine plant genomes indicated that the pistachio diverged from *Citrus sinensis* ~ 58 million years ago and from *Populus trichocarpa* ~ 105 million years ago (Additional file 1: Figure S1). Analysis of fourfold degenerate third-codon transversion sites demonstrated that the pistachio genome had not experienced a lineage-specific whole genome duplication subsequent to its divergence from these species (Additional file 1: Figure S2). We also performed a genomic synteny analysis by aligning the pistachio genome to the genome of the basal angiosperm *Amborella trichopoda* [7]. The macrosynteny analyses showed that each *Amborella* region had up to three pistachio regions, while each pistachio region had up to two *P. trichocarpa* regions [8] (Fig. 1b, Additional file 1: Figure S3). The synteny analyses support the conclusion that no lineage-specific genome duplication occurred in pistachio, but they do share the gamma duplication that occurred within eudicots, and that *Populus* experienced a lineage-specific genome duplication event [7, 8].

### Expanded gene families related with stress adaptation of pistachio

To reveal the genetic basis underpinning the pistachio phenotype (e.g., salt tolerance), we investigated the evolution of gene families by identifying unique and shared gene families among different plants using OrthoMCL [9]. Among the gene families identified in *P. vera*, 9735 were shared as families with *Arabidopsis thaliana*, *C. sinensis*, *Gossypium raimondii*, and *Vitis vinifera*, while 707 gene families, containing 1381 genes, were specific to pistachio (Additional file 1: Figure S4). To assess the function of these genes, we performed a Gene Ontology (GO) and Kyoto Encyclopedia of Genes and Genomes (KEGG) enrichment analysis using David and g:Profiler programs. Both programs found many genes involved in “defense response” (GO: 0006952, Additional file 2: Table S7), which included many genes containing the NB-ARC domain and the NBS-LRR domain. This kind of genes has been well known for disease resistance in plants [10] and is reasonably important for defense response in pistachio.

Next, we examined the expansion and contraction of gene families in pistachio (Fig. 1c). As it is difficult to reach conclusions concerning adaptation from contraction in gene family size, or with genes that were not successfully assembled in this reference genome [11], we only analyzed the expanded gene families. Gene enrichment analysis of the expanded gene families found them to be enriched in the categories of metabolism, such as biosynthesis of terpenoid, flavonoid, sesquiterpenoid, and alkaloid (Additional file 2: Table S8). The expansion of gene families occurs after a long-term evolution and drives the evolutionary difference between *Pistacia* and *Citrus*, rather than a very short-term evolution of pistachio domestication from the wild. Therefore, we propose that the expansion of genes in the above categories is probably related to the metabolism of organic compounds found in wild *Pistacia* species. Phytochemical screening of wild *Pistacia* species found many phytochemicals such as alkaloids, flavonoids, coumarins, sterols, tannins, terpenoids, and sesquiterpene [12–14].

In addition, the enriched term “oxidation-reduction process” (GO: 0055114, *P* ≪ 0.001) contains many cytochrome P450 genes, which encode proteins involved in multiple metabolic pathways with complex functions and playing important roles in multiple processes, particularly roles in stress responses. To assess the function of these genes, we used BLASTP to search the *A. thaliana* proteome and identified the best-hit genes (*E* value < 1e−10) (Additional file 2: Table S9). Among the 187 cytochrome P450 genes, we found that many probably had functions for salt tolerance. For example, pervious study found elevated levels of *CYP94* family gene expression alleviate the jasmonate response and enhance salt tolerance in rice [15]. Among these expanded gene families in pistachio, there are 14 members of *CYP94* genes. In soybean, *CYP82A3* is involved in the jasmonic acid and ethylene signaling pathway and enhances resistance to salinity and drought [16], and there are 20 members of *CYP82* genes among the expanded gene families in pistachio. Ectopic expression of *P. trichocarpa CYP714A3* enhanced salt tolerance in rice [17], and there are 10 members of *CYP714A* genes among the expanded gene families in pistachio. Thus, it is likely that some cytochrome P450 genes are responsible for salt tolerance in pistachio.

### RNA sequencing reveals a genetic mechanism underlying salt adaptation of pistachio

**Fig. 2:**
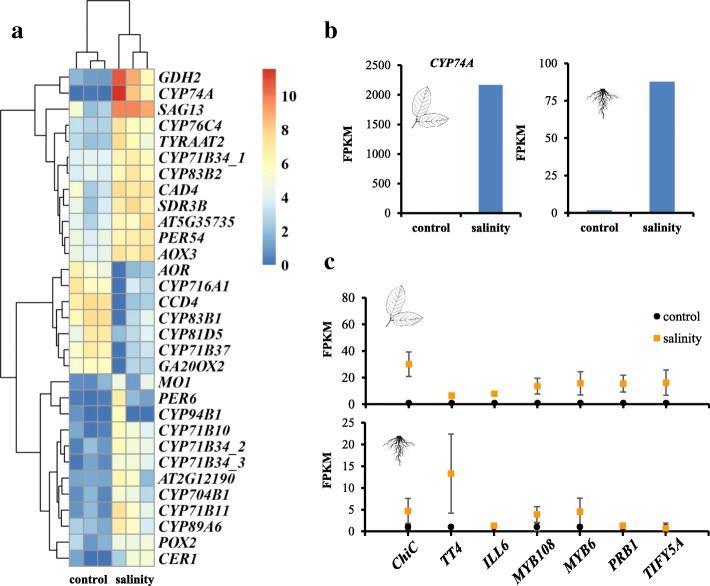
Transcriptome analysis of saline-treated pistachio. **a** Expression heatmap of genes in the “oxidation-reduction process”. C, control pistachio leaf; S, saline-treated pistachio leaf. *n*_control_ = 3, *n*_salinity_ = 3. Expression values were normalized by log2 (FPKM+1). **b** Expression values (FPKM) of *CYP74A* in the leaf (left) and root (right). **c** Relative expression levels of seven genes in the category “response to jasmonic acid”

To further investigate the genetic mechanisms underlying salt tolerance in pistachio, we performed a salinity experiment. Leaf and root transcriptomes of pistachio rootstock, *P. vera L.* cv. Ohadi, grown under normal and salinity conditions (see the “Methods” section) were generated by RNA sequencing. Using the Tophat-Cufflinks-Cuffdiff pipeline [18], 214 and 461 protein-coding genes were identified to exhibit differential expression, respectively in leaf and root tissues (*n*_control_ = 3, *n*_salinity_ = 3, corrected *P* < 0.05, Additional file 1: Figure S5), between plants treated under saline conditions versus the control. Gene enrichment analysis found that many of the differentially expressed genes (31 genes) are involved in “oxidation-reduction process” (Fig. 2a, b; Additional file 2: Tables S10-S11). As in the comparative genomic analysis, 15 genes in this category are cytochrome P450 genes, specifically, *CYP74A* (i.e., *AOS*), which encodes a member of the cytochrome P450 CYP74 gene family that functions as an allene oxide synthase (AOS). This enzyme catalyzes the first committed step in the synthesis of jasmonates (i.e., jasmonic acid (JA)) [19]. The expression fragments per kilobase of exon per million fragments mapped (FPKM) values for *AOS* increased from nearly 0 in the control to 2163.75 under saline conditions in the leaf, and from 1.87 in the control to 87.74 for the saline-treated root (Fig. 2c). We also found that 7 differentially expressed genes (*ChiC*, *TT4*, *ILL6*, *MYB108*, *MYB6*, *PRB1*, and *TIFY5A*) were enriched for “response to jasmonic acid” (*P* = 0.005 after correction; Fig. 2c, Additional file 2: Tables S10-S11). Previous studies have shown that both drought and high salinity caused increased JA levels in the leaves and roots of rice [20, 21]. Salinity treatment can increase endogenous JA level in the *Iris hexagona*, a wetland species [22]. Jasmonates activate plant responses to biotic stresses (i.e., attack by pathogens) and abiotic stresses (i.e., salt) [23]. Here, expression levels of these genes involved in response to jasmonic acid are increased in the leaf and root with the saline treatment (Fig. 2c). The increased expressions of these genes (e.g., AOS as enzyme catalyzing the first committed step in the synthesis of jasmonates) should increase the synthesis of jasmonates, and thus, they are likely used by pistachio to respond to salt stress.

Differentially expressed genes were also found to be enriched in “chitin binding” (*P* = 0.03 after correction, Additional file 2: Table S10), with four genes encoding chitinases (*CHIB*, *EP3*, *ChiC*, *AT2G43590*). Plant chitinases are involved in diverse biological systems. Some chitinases in plants are expressed in response to environmental stresses (i.e., high salt concentration, cold, and drought) and can be upregulated by phytohormones such as ethylene, jasmonic acid, and salicylic acid [24, 25]. For example, gene *ChiC* encodes a class V chitinase, and its expression can be induced by the jasmonic acid and the stress resulting from salinity in *Arabidopsis thaliana* [26]. *CHIB* encodes a basic chitinase involved in jasmonic acid-mediated signaling pathway [27]. Our transcriptomic analysis suggests that genes encoding chitinases and those involved in the JA biosynthetic pathway likely contribute to the adaptation of pistachio to saline environments.

### Admixture occurred among different wild relatives

**Fig. 3:**
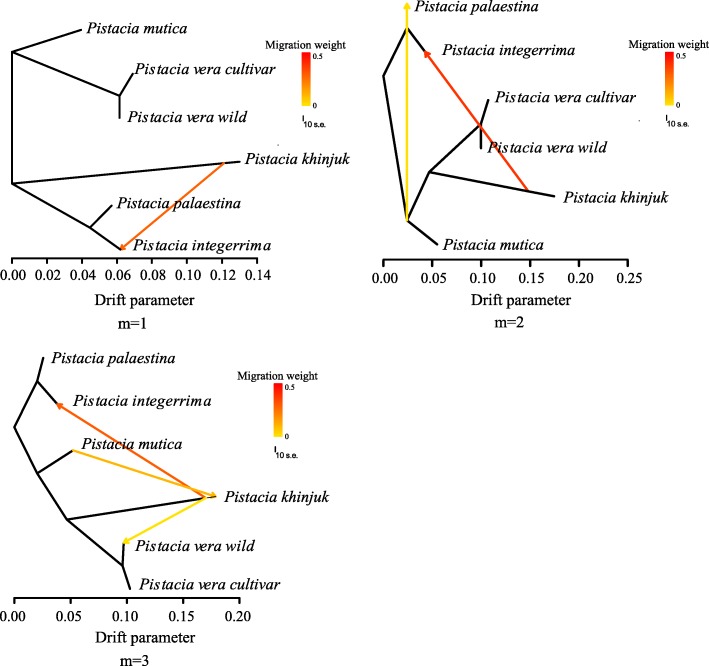
Signal of introgression among different wild species detected by the TreeMix program. Hybridization likely occurs among the different close relatives in nature. However, no introgression was detected from other wild species to cultivated pistachio

To investigate the demographic history and adaptive evolution of pistachio, we resequenced an additional 107 genomes from *P. vera* including 93 cultivars and 14 genomes of wild pistachio to an average depth of 6~8×. We also resequenced 35 genomes from different close species, including *P. mutica*, *P. khinjuk*, *P. integerrima*, and *P. palaestina* (Additional file 2: Tables S12-S13). Using a stringent GATK pipeline [28], 14.77 million single-base variants were called, with 2.42 million of them being in genic regions (intronic and exonic; 412,917 nonsynonymous, 354,937 synonymous) (Additional file 2: Tables S14-S17, Additional file 1: Figure S6-S9). Phylogenetic analyses using the neighbor joining and maximum likelihood methods clearly separated the 5 different species, i.e., *P. vera*, *P. mutica*, *P. khinjuk*, *P. integerrima*, and *P. palaestina* (Additional file 1: Figure S10-S11). Signals of introgression were detected between some species by the TreeMix program [29], i.e., from *P. khinjuk* to *P. integerrima* (Fig. 3), which were supported by the ABBA-BABA test (Additional file 1: Figure S12). This indicates that hybridization likely occurs among the different close relatives in nature and is consistent with the pervasive hybridization seen in plants [30, 31]. However, no introgression was detected from other pistachio species to domesticated pistachio, which was derived from wild *P. vera*.

### A two-step domestication of pistachio

**Fig. 4:**
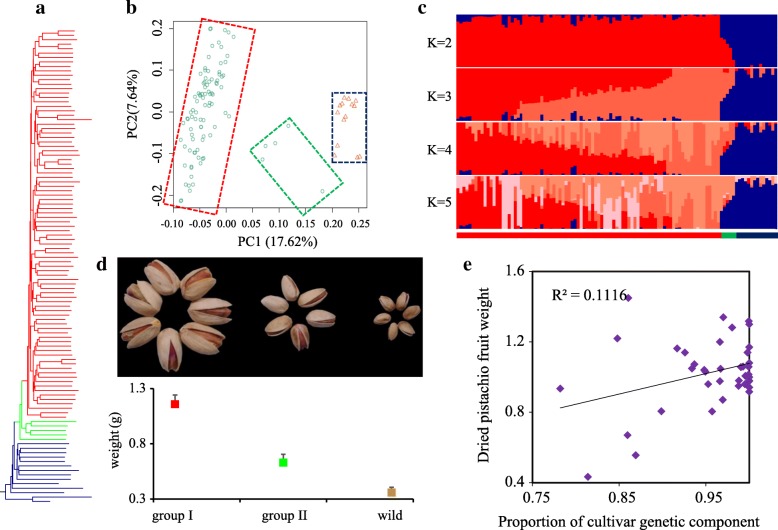
Phylogenetic analysis of wild and domestic pistachio. **a** Phylogenetic tree. Cultivars of group I (red), cultivar group II (green), and wild pistachio (blue) are marked in different colors from top to bottom. **b** PCA analysis. From left to right, the three squares indicate cultivar group I, cultivar group II, and wild pistachio. **c** Admixture analysis. From left to right, the three groups, i.e., cultivar group I, cultivar group II, and wild pistachio are marked by different colors. **d** From left to right, representative dry seeds from cultivar group I, cultivar group II, and wild pistachio. **e** Correlation between the proportion of cultivar genetic component and dried pistachio fruit weight

Based on these resequenced genomes, we inferred the changes in effective population sizes of these species and found a bottleneck event during the Pleistocene period and an increase in their effective population size ~ 200 kyr ago (Additional file 1: Figure S13). Our phylogenetic tree shows a clear separation between domestic and wild pistachio (Fig. 4a). The divergence time between wild and domestic was inferred by δaδi to be ~ 8000 years ago (Additional file 1: Figure S13, Additional file 2: Table S18), which is similar to the archeological record showing that pistachio seeds were a common food as early as 6750 BC [32]. To gain insight into the genetic relationships among the pistachio accessions, we performed two classical analyses: population structure and principal component analysis (Fig. 4b, c; Additional file 1: Figure S14). These analyses clearly show two groups of cultivar accessions. The level of linkage disequilibrium (LD) is highest within cultivar group I, while the rate of decay of LD is nearly the same in both cultivar group II and wild pistachio (Additional file 1: Figure S15). Group II includes five individuals from the cultivars Qazvini, Italiaei, and Badami Zarand, which are recorded to be ancient and harboring seeds of small size (Fig. 4d). Consistent with the phylogenetic tree, these three cultivars also contained a higher proportion of wild ancestry (Fig. 4e), which supports a two-step domestication processes, with initial domestication, followed by improvement through crop breeding.

### Genetic mechanisms underlying pistachio domestication

**Fig. 5:**
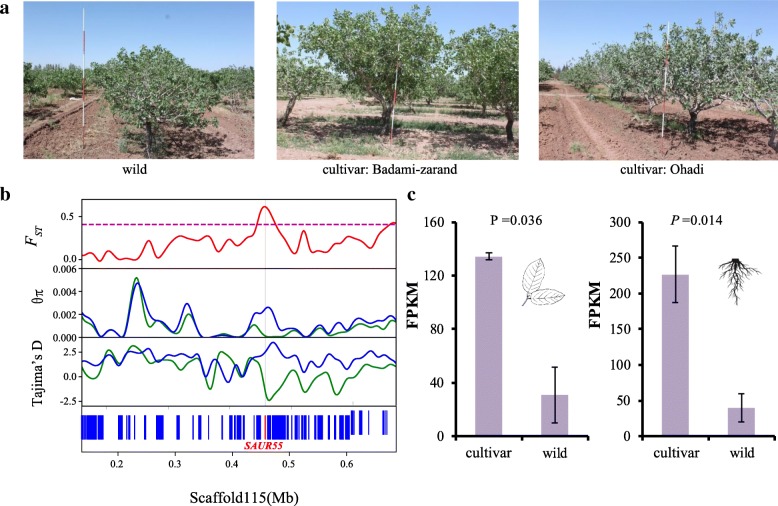
Artificial selection on pistachio tree size. **a** From left to right are the photographs of trees of wild pistachio, Badami-zarand cultivar, and Ohadi cultivar; the trees are of the same age and grown under similar conditions at the Pistachio Research Center, Rafsanjan, Iran. The same marker post is located beside each of the trees. **b** *F*_ST_, *θπ*, and Tajima’s *D* value of *SAUR55* between cultivated and wild pistachio. **c** Relative expression levels of *SAUR55* in the leaf and root, *n*_cultivar_ = 3, *n*_wild_ = 3

Average pairwise nucleotide diversity within populations (*θπ*) indicated lower diversity among the domestic accessions compared with the wild (Additional file 2: Table S19). Using a log2 ratio of *θπ* between cultivated and wild samples, we found some genomic regions that showed reduced diversity in the cultivated that may contain genes under artificial selection (Additional file 1: Figure S16). In addition, we identified regions with an increased level of differentiation (*F*_ST_) between the cultivated and wild samples (Additional file 1: Figure S16-S17). About 9.2 Mb of genomic regions were identified to display high levels of population differentiation between domestic and wild pistachio, and low levels of genetic diversity among cultivars, being above the 95% threshold for both properties (Additional file 1: Figure S16, Additional file 2: Tables S20-S21). Regions of reduced diversity and enhanced population differentiation might have experienced selective sweeps during domestication or breeding. In total, 665 genes were located in these regions (Additional file 2: Tables S22-S24). We focused on candidate positively selected genes that might be associated with the evolution of phenotypes important for domestication. The tree size was a target for artificial selection during the domestication of pistachio (Fig. 5a). We found the gene *SAUR55* (Fig. 5b), encoding auxin response protein that plays important roles in plant growth [33], evolved under artificial selection in pistachio. Particularly, gene *SAUR55* displays a significantly increased expression level in domestic compared to wild pistachio, based on the leaf and root transcriptome data (Fig. 5c). These observations are consistent with evidence of selective sweeps on auxin response genes in other crops, such as rice [34] and wheat [35], and reveal convergent artificial selection for similar traits during crop domestication. Fruit weight is among the most important traits targeted during domestication and breeding of crops, including pistachio. A positive correlation was found between the proportion of cultivar component and fruit weight among cultivars (Fig. 4e). This supports the conclusion that artificial selection on fruit weight occurred on pistachio during domestication and cultivation. We note that the gene *CYCD7-1* evolved under artificial selection with a signature of a high level of population differentiation between wild and domestic cultivars (Additional file 1: Figure S18-S19). This gene encodes a D-type cyclin, which controls cell division and growth rate during seed development. Over-expression of *CYCD7-1* induces cell proliferation and cell enlargement in the embryo and endosperm, leading to the overgrowth of seed, in *Arabidopsis* [36]. Gene *CYCD7-1* displays special expression in pollen and early development, but no expression in the leaf and root (Additional file 1: Figure S20). Therefore, it is promising to compare expression of *CYCD7-1* in pollen and early development of wild and domestic pistachio in the future experiment. We propose that artificial selection on *CYCD7-1* might occur to improve pistachio fruit weight.

## Conclusions

In this study, we assembled a draft reference genome and used comparative genomics to reveal the genetic underpinning for salt tolerance adaptation in pistachio, one of the most important commercial nut crops cultivated in desert regions. This reference genome will enable future evolutionary and ecological research on pistachio. We also generated a genome-wide dataset of SNP from 93 domestic and 14 wild individuals and identified some genes for agronomically related traits, such as fruit weight and tree size, that might have been selected during the domestication of pistachio. Sequence information from diverse cultivar accessions should be helpful for the researchers and breeders in genome-wide association mapping studies of agronomically related traits and support marker-assisted and genomic selection-based approaches for plant improvement. Insight into the environmental adaptations and economic characters of this crop will undoubtedly facilitate planting and breeding programs for different desert regions, which might contribute to easing the world’s food crisis.

## Methods

### Illumina sequencing for de novo genome

An individual of *P. vera*, cultivar name Batoury, a variety widely cultivated in China, was chosen for genome sequencing and assembly. Genomic DNA was extracted using the DNeasy Plant Mini Kit (Qiagen). Multiple pair-end libraries, including two types of small-insert libraries (270 bp and 500 bp) and six types of long-insert mate-pair libraries (3 kb, 4 kb, 8 kb, 10 kb, 15 kb, and 17 kb), were constructed using the Illumina paired-end and mate-pair kits according to the manufacturer’s instructions.

The libraries were sequenced on the Illumina Hiseq 2500 platform. For the raw reads, sequencing adaptors were removed and contaminated reads (chloroplast, mitochondrial, bacterial and viral sequences, etc.) were screened by alignment to the NCBI NR database using BWA v0.7.13 [37] with default parameters. FastUniq v1.1 [38] was used to remove duplicated read pairs, and low-quality reads were filtered under the following conditions: (1) reads with ≥ 10% unidentified nucleotides (*N*); (2) reads with > 10 nucleotides aligned to the adapter, allowing ≤ 10% mismatch; and (3) reads with > 50% bases having Phred quality < 5. Finally, we generated a total of 98.08 Gb and 55.87 Gb clean reads for the paired-end and the mate-pair libraries, respectively.

### PacBio sequencing and assembly

Single-molecule sequencing was performed by the PacBio Sequel System, which yielded a total of 4,038,150 filtered subreads with average lengths of 14,568 bp for *P. vera*. Finally, only equal or longer than 500 bp PacBio subreads were performed the gap filling. Based on the ALLPATH-LG assembly, we utilized PBJelly [39] to do gap filling with the filtered PacBio subreads for improving genome assembly, and the option is “<blasr> -minMatch 8 -minPctIdentity 70 -bestn 1 -nCandidates 20 -maxScore -500 -noSplitSubreads </blasr>.” The final polishing procedure was completed by pilon v1.22 (available at https://github.com/broadinstitute/pilon) using Illumina data with the parameters “--mindepth 10 --changes --threads 4 --fix bases.”

### Genome size estimation

A total of 58.80 Gb Illumina reads from the 270 bp library were selected to perform the genome size estimation. The distribution of 21-*k*mer showed a major peak at 84×. Based on the total number *k*mers and the corresponding *k*mer depth of 84, the pistachio genome size was estimated to be ~ 519.17 Mb using the formula: Genome size = *k*mer_Number/Peak_Depth.

### De novo assembly and assessment

The whole genome was de novo assembled into longer contigs using ALLPATH-LG [40] with the default parameters. Because high genomic heterozygosity is generally recovered as alternative contigs, REDUNDANS v.013b [41] was used to identify heterozygous contigs based on at least 85% identity and overlap of at least 66% of the shorter sequence length from each pairwise comparison, with only the longer of the two redundant contigs retained. Finally, adjacent contigs connected by mate-pair information were joined to scaffolds using SSPACE v2.3 [42] and gap filling was proceeded by GapCloser v1.12 from the SOAPdenovo package [43].

After detecting the variation using the corresponding sequencing data, the SNP rate besides the genome heterozygosity was estimated as the error rate of the genome assembly. Completeness of the assembly was assessed by mapping 458 conserved core eukaryotic genes (CGEs) and 248 highly conserved CGEs to the genome using CEGMA v2.5 [44].

### Genome annotation

#### Repetitive sequences

The amount of the assembly composed of repeats was estimated by building a repeat library employing the de novo prediction programs LTR-FINDER [45], MITE-Hunter [46], RepeatScout v1.0.5 [47], and PILER-DF [48]. The new repetitive elements were classified using PASTEClassifier v1.0 [49] and combined with Repbase database v20.01 [50] to create the final repeat library. Repeat sequences in the pistachio genome were identified and classified using the RepeatMasker program v4.0.6 [51]. The criterion used for LTR family classification was that the 5′LTR sequence would share at least 80% identity over at least 80% of their length for the same family.

#### Protein-coding genes

Protein-coding genes were predicted using de novo and protein homology-based approaches. Genscan v1.0 [52], Augustus v2.5.5 [53], GlimmerHMM v3.0.1 [54], GeneID v1.3 [55], and SNAP [56] were performed for de novo gene prediction, while homologous peptides from the *A. thaliana* (TAIR 10), *Oryza sativa* (Nipponbare, IRGSP-1.0), *Theobroma cacao* (Phytozome v12.1), and *C. sinensis* (Phytozome v12.1) genomes were aligned to our assembly to identify the homologous genes with GeMoMa v1.4.2 [57]; the RNA-Seq reads were assembled into contigs de novo into unigenes using Trinity [58], and the resulting unigenes were aligned to the repeat-masked assemblies using BLAT [59], and subsequently, the gene structures of BLAT alignment results were modeled using PASA [60]; then, the protein-coding regions were identified with TransDecoder v3.0.1 [61] and GeneMarkS-T [62], respectively. Consensus gene models were generated by integrating the de novo predictions and protein alignments using EVidenceModeler [63]. Annotation of the predicted genes was performed by blasting the gene (and predicted protein) sequences against a number of nucleotide and protein sequence databases, including COG [64], KEGG [65], NCBI-NR, and Swiss-Prot [66] with an *E* value cutoff of 1e−5 used to assess orthology.

#### Non-coding RNAs

rDNA genes were identified by aligning with rRNA template sequences (Pfam database v22.0 [67], using BLAST [68] with an *E* value of 1e−10 and an identity cutoff of 95% or more. The tRNAScan-SE v2.0 [69] algorithm with default parameters was applied to predict tRNA genes. miRNA genes were predicted using INFERNAL v1.1 software [70] with the Rfam database v11.0 [71] and a cutoff score of 30 or more. The minimum cutoff score was based on the settings which yielded a false-positive rate of 30 bits.

### Comparative analysis of gene families

To identify homologous relationships among pistachio and related plants, the pistachio proteome was globally compared with the *A. thaliana*, *C. sinensis*, *G. raimondii* (Phytozome v12.1), and *V. vinifera* (Phytozome v12.1) proteomes filtered for transposable elements and alternative splicing. An all-against-all comparison was performed using BLASTP (*E* value = 1e−5) followed by clustering with OrthoMCL v2.0.9 [9] (inflation = 1.5). Analysis of species-specific gene families was made with a Fisher’s exact test (*P* < 0.0001) on the Pfam domains.

### Phylogenomic analysis

A phylogenetic tree of 9 species—*V. vinifera*, *Eucalyptus grandis* (Phytozome v12.1), *G. raimondii*, *Medicago sativa* (Phytozome v12.1), *Brassica rapa* (Phytozome v12.1), *A. thaliana*, *P. trichocarpa* (http://ensemblgenomes.org,release-21), *C. sinensis*, and pistachio—was constructed using PhyML software v3.0 [72] based on 1096 shared single-copy genes. The divergence time between pistachio and the 8 other sequenced species (*V. vinifera* as the outgroup) was estimated using the MCMCtree program implemented in the PAML package v4.9 [73]. Calibration times were obtained from the TimeTree database (http://www.timetree.org/). Expansion and contraction of OrthoMCL-derived gene clusters was determined by a CAFÉ v2.1 [74] calculation on the basis of changes in gene family size in the inferred phylogenetic history. KEGG and GO annotation of gene family was completed by aligning the genes to the KEGG database and NCBI non-redundant database using BlastP with an *E* value of 1e−5, respectively. BLAST2GO [75] was used to obtain the associated GO terms. The enrichment score is defined as the hypergeometric test value [76].

### Whole genome duplication analysis

The all-against-all BLASTP method (*E* value < 1e−5) was used to detect paralogous genes in pistachio, *C. sinensis*, *V. vinifera*, and *P. trichocarpa* as well as orthologous genes in pistachio *C. sinensis* and pistachio *V. vinifera*. Homologous blocks were then detected using Mcscan v1.1 [77]. The synonymous substitution (Ks) and fourfold degenerate site transversion (4DTV) values of the blocks were calculated using the HKY model [78].

Syntenic depth refers to the number of times a genomic region (or genome) is syntenic to the regions in another genome [79]. We performed synteny searches to compare the pistachio genome structure with *A. trichopoda* (*ε*-WGD [7]) (http://amborella.huck.psu.edu,version1.0) and *P. trichocarpa* (*ε*-WGD, γ-WGD, and β-WGD [8]) genomes.

### Salinity experiment

A greenhouse experiment was conducted in 2016 at the Pistachio Research Center, Rafsanjan, Iran: 30° 24′ 49.0″ N, 55° 59′ 20.8″ E, at 1528 masl. Seeds of cultivar pistachio rootstocks, *P. vera L. cv*. Ohadi, and the wild type (Sarakhs) were surface sterilized with 5% solution of sodium hypochlorite in distilled water and then incubated at 30 °C on a sterile moist cloth for 1 week. Ten seeds that germinated were sown in each pot filled with about 7 kg sandy-loam soil at a depth of about 2 cm. The number of seedlings per pot was reduced to three uniform seedlings when the emergence period was completed about 3 weeks of planting.

The experiment consisted of a completely randomized design with three replications for a total of six pots. Irrigation of all pots was carried out for 2 months using Rafsanjan normal urban water (with a salinity of 0.6 dS m^−1^) at field capacity. Two months after planting, when seedlings were well established, salinity treatment was imposed on half of the pots (salinity group) by irrigation water with an electrical conductivity of 4.5 dS m^−1^ while the other half of the pots (control group) were irrigated using the Rafsanjan normal urban water throughout the entire experiment. Salinity stress continued for 1 month when the symptoms of salinity toxicity in the leaves (burning edge caused by salt toxicity) were observed. Leaf samples were taken before applying the salinity stress on the seedlings at 2 months of age. Both the leaf and root samples from all replicates were taken at the end of the experiment when the seedlings were 3 months of age. The fresh leaf and root tissue samples were immediately dissected and submerged in five volumes of RNA*later*® solution and stored in a − 80 freezer for subsequent RNA extraction. During the experiment, the maximum temperature was 34 ± 5 °C; the minimum temperature was 21 ± 4 °C, and the relative humidity was 40 ± 5%.

### RNA sequencing

The leaves and roots from the three salinity-treated samples of cultivar, three samples under control condition of cultivar, and three samples under control condition of wild were prepared for RNA sequencing. Total RNA was extracted using the RNAplant Plus Reagent according to the manufacturers’ instructions (Tiangen, Beijing, China). Before library construction, we evaluated the degradation of the RNA on a 1% agarose gel and checked the purity using the Qubit® 3.0 Fluorometer (Life Technologies, CA, USA), with integrity and concentration assessed using an RNA Nano 6000 Assay Kit on the Bioanalyzer 2100 system (Agilent Technologies, CA, USA). Sequencing libraries were generated using NEBNext® Ultra™ RNA Library Prep Kit for Illumina® (#E7530L, NEB, USA) following the manufacturer’s recommendations and then sequenced on the Illumina HiSeq X Ten platform to generate 150 bp paired-end reads.

Before alignment, reads were trimmed based on their quality scores using the quality trimming program Btrim [80]. Reads were aligned to our de novo genome of *Pistacia vera* L. using TopHat (v2.1.1 [18]) and then assembled using Cufflinks (v2.2.1 with –G parameter) [18]. Differential expression of genes in the different tissues was calculated using Cuffdiff [18].

### Phenotyping

Fruit size-related traits for pistachio were measured based on the pistachio descriptor (IPGRI. 1997. Descriptors for pistachio (*P. vera L.*), International Plant Genetic Resources Institute, Rome, Italy). The following phenotypes were recorded: dried pistachio fruit weight (gr) (the mean weight of 100 healthy dry nuts), fresh fruit weight with green skin (gr) (the mean weight of 100 healthy fresh nuts with green skin in grams), dried pistachio fruit length (mm) (average of 20 healthy nuts, measured from the most distant points along the main seed axis), dried pistachio fruit diameter (mm) (average of 20 healthy nuts, measured at the widest part perpendicular to the suture), dried pistachio fruit width (mm) (average of 20 healthy nuts, measured from the widest points perpendicular to the main seed axis), dried pistachio fruit and kernel shape (roundish = 1, ovoid = 2, elongated = 3, narrowly cordate = 4, cordate = 5), dried kernel weight (gr) (average weight of 100 healthy dry kernels), kernel diameter (mm) (average of 20 healthy kernels, measured at the widest part perpendicular to the cotyledon suture.), kernel width (mm) (average of 20 healthy kernels, measured on the widest points perpendicular to the main seed axis), kernel length (mm) (average of 20 healthy kernels, measured from the most distant points along the main seed axis).

### Plant material and DNA extraction for genome resequencing

Fresh leaves (4–5 g) were sampled from the germplasm collections of the Pistachio Research Institute in Rafsanjan, Iran: 30° 24′ 49.0″ N, 55° 59′ 20.8″ E, at 1528 masl; the pistachio germplasm of Ardakan, Iran: 32° 18′ 36″ N, 54° 1′ 3″ E, at 1040 masl; and Jiroft, Iran: 28° 40′ 41″ N, 57° 44′ 26″ E, at 650 masl. Leaf tissues were harvested during the 2015–2017 period and were transported on ice and stored at − 80 °C in the Biotechnology Laboratory, Animal Science Department, Shahid Bahonar University of Kerman, Iran, until subjected to DNA extraction.

Total genomic DNA was extracted from 1 g fresh leaves using hexadecyl trimethyl ammonium bromide (CTAB) protocol with some modifications. The quantity and quality of isolated DNA were assessed by NanoDrop spectrophotometer and 1% agarose gel electrophoresis, looking for a single absorbance peak at 260 nm, a 260/280 absorbance ratio of 1.8–2.0, and no evidence of substantial band shearing or contamination. Extracted DNA was dissolved in 20 μl TE buffer (10 mM Tris, pH 8, 1 mM EDTA) and stored at − 20 °C for subsequent NGS analysis.

### Genome resequencing and SNP calling

Among the above samples, 93 domestic and 14 wild pistachio individuals and another 13 *P. mutica*, 13 *P. khinjuk*, 4 *P. integerrima*, and 5 *P. palaestina* were chosen for genome resequencing. Ten micrograms of genomic DNA, prepared by the standard CTAB extraction protocol, was used to construct libraries with 350 bp insert size. Sequence libraries were constructed according to the Illumina library preparation pipeline and were sequenced on the Illumina Hiseq 4000 platform to generate 150 bp paired-end reads.

We mapped the reads to our reference genome (version 1) with BWA-MEM [81]. After sorting and duplicate marking of the bam format files with Picards tools 1.56 (http://picard.sourceforge.net), we called SNPs using Genome Analysis Toolkit (GATK) [28]. The criteria used to filter the raw SNPs were “QUAL < 40.0, MQ < 25.0, MQ0 >= 4 && ((MQ0/(1.0*DP)) > 0.1, -cluster 3 -window 10.” We ignore the multi-nucleotide polymorphisms, and the loci containing SNP markers must present in at least 90% of individuals. A total of 14,767,700 high-quality SNPs were identified and used in the subsequent analyses.

### Phylogenetic relationship of resequenced pistachio

We constructed a neighbor-joining tree using a *p*-distance matrix by TreeBeST (http://treesoft.sourceforge.net/treebest.shtml) with 100 bootstrap replications. Principal component analysis (PCA) was performed using the toolset SNPRelate from the R package. We ran frappe to estimate the genetic ancestry of each sample, specifying a range of 2–5 hypothetical ancestral populations. The maximum number of interaction was set to 10,000 in the frappe analysis.

### Artificial selection analysis

We calculated the genome-wide distribution of population fixation statistics *F*_ST_ [82] and nucleotide diversity *θπ* and Watterson’s estimator *θw* ratios (windows with a number of variants < 20 were ignored) [83] for each sliding window with a window size of 50 kb and a step size of 25 kb. Putative selection targets were extracted with both top 5% of log ratios for *θπ* and *F*_ST_. Our approach was to identify genomic regions with high differentiation between cultivated and wild *Pistacia vera* (*n*_cultivar_ = 13, *n*_wild_ = 14).

### Analysis of genetic introgression

We inferred gene flow between the diverged populations using the maximum likelihood method implemented in TreeMix [29]. First, we inferred the maximum likelihood (ML) tree with the command “-i input -bootstrap -o output.” Second, from one to four migration events were gradually added to the ML tree of the five species with command was “-i input -bootstrap -k 1000 -m migration events -o output.” Genetic introgression was also analyzed by the *D* statistic (ABBA-BABA test) [84].

### Recent demographic history inference using δaδi

To uncover the recent demographic history of the cultivated and wild *P. vera*, we only considered SNPs with more than 40-fold sequencing coverage at the population level in intergenic regions to ensure their neutrality. Missing genotypes were imputed using the program BEAGLE [85]. After investigating the empirical distributions of the MAFs, haplotypes were inferred for all genotype sites with MAF > 0.01. Two divergence models were considered between the two populations of *P. vera*. The model with the maximum log-likelihood value was chosen as the optimal one.

### PSMC analysis

Dynamic changes in the effective population size of *Pistacia* species were inferred using the PSMC program [86], with a mutation rate of 7.7e−9 and a generation time of 10 years [87].

### GO and KEGG enrichment analysis

Gene Ontology (GO) enrichment analyses were performed using the DAVID program (https://david.ncifcrf.gov/) and g: profiler (https://biit.cs.ut.ee/gprofiler/).

## Additional files

Additional file 1: **Figure S1-S20.**. Supplementary figures supporting the manuscript. (DOCX 5084 kb)

Additional file 2: **Tables S1-S24.** Supplementary tables supporting the manuscript. (XLSX 281 kb)
