# Supplementary material for: Whole genomes and transcriptomes reveal adaptation and domestication of pistachio
Source: Genome Biol. 2019 Apr 18;20:79. doi: 10.1186/s13059-019-1686-3 (PMC6474056; doi:10.1186/s13059-019-1686-3)
Supplement: Supplementary file 1 — Figure S1-S20.. Supplementary figures supporting the manuscript. (DOCX 5084 kb) [file 13059_2019_1686_MOESM1_ESM.docx]

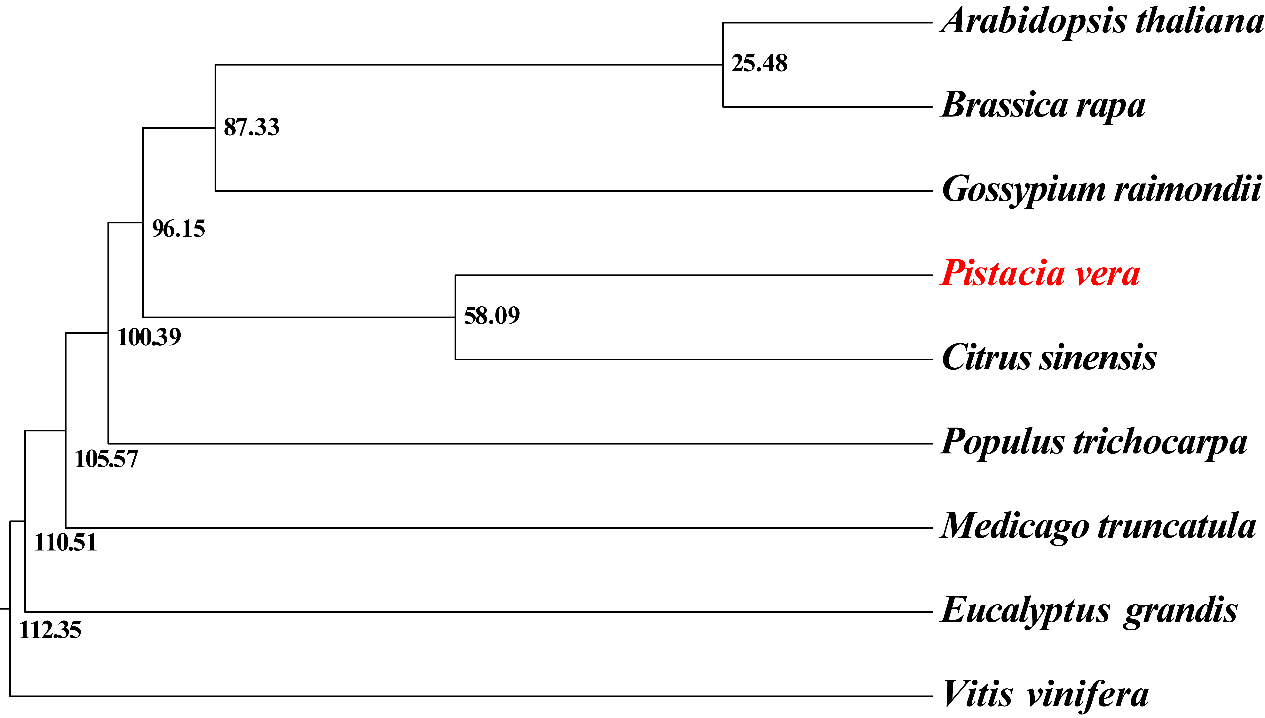


**Figure S1:** Phylogenomic analysis of the divergence times between pistachio and eight other plant species using PhyML software based on 1,096 shared single copy genes. Pistachio diverged from *C. sinensis* ~58 million years ago and from *P. trichocarpa* ~ 105 million years ago.


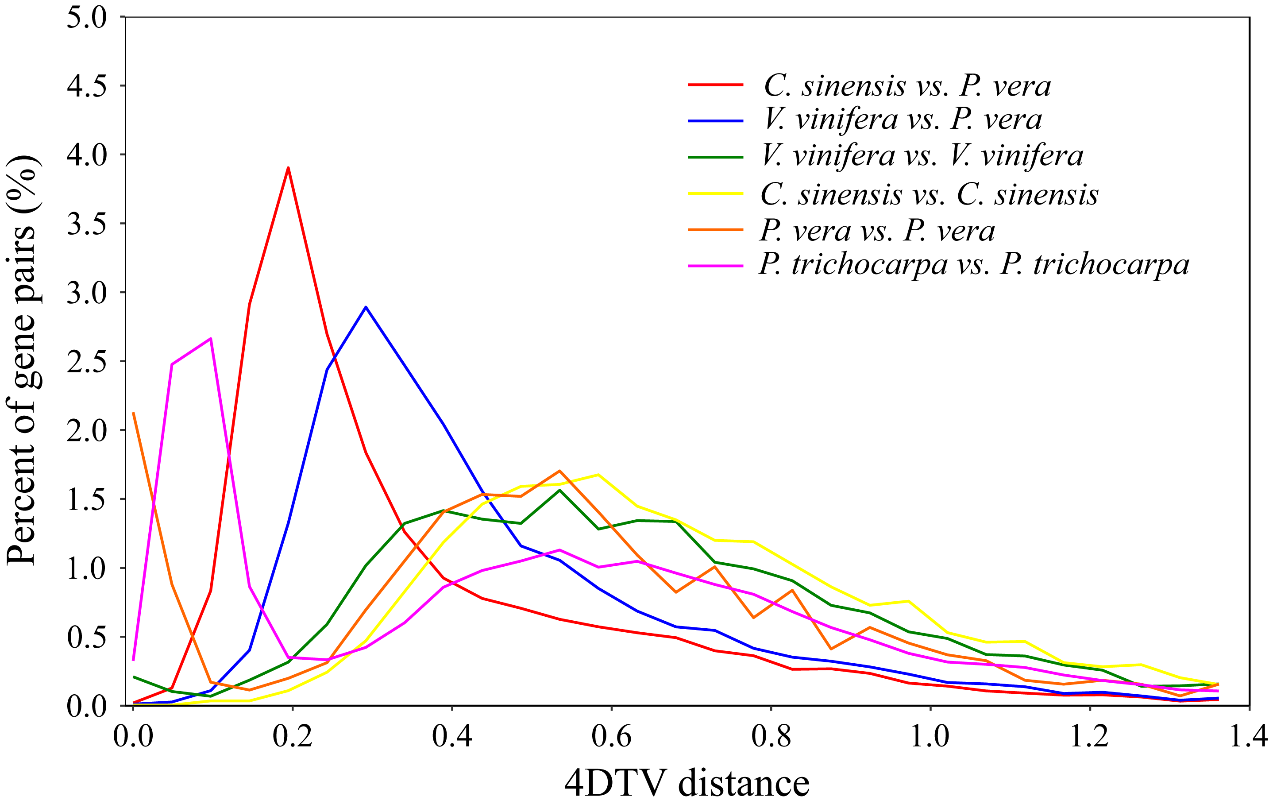


**Figure S2:** Analysis of fourfold degenerate third-codon transversion sites demonstrated that the pistachio genome had not experienced a lineage-specific whole genome duplication subsequent to its divergence from these species.


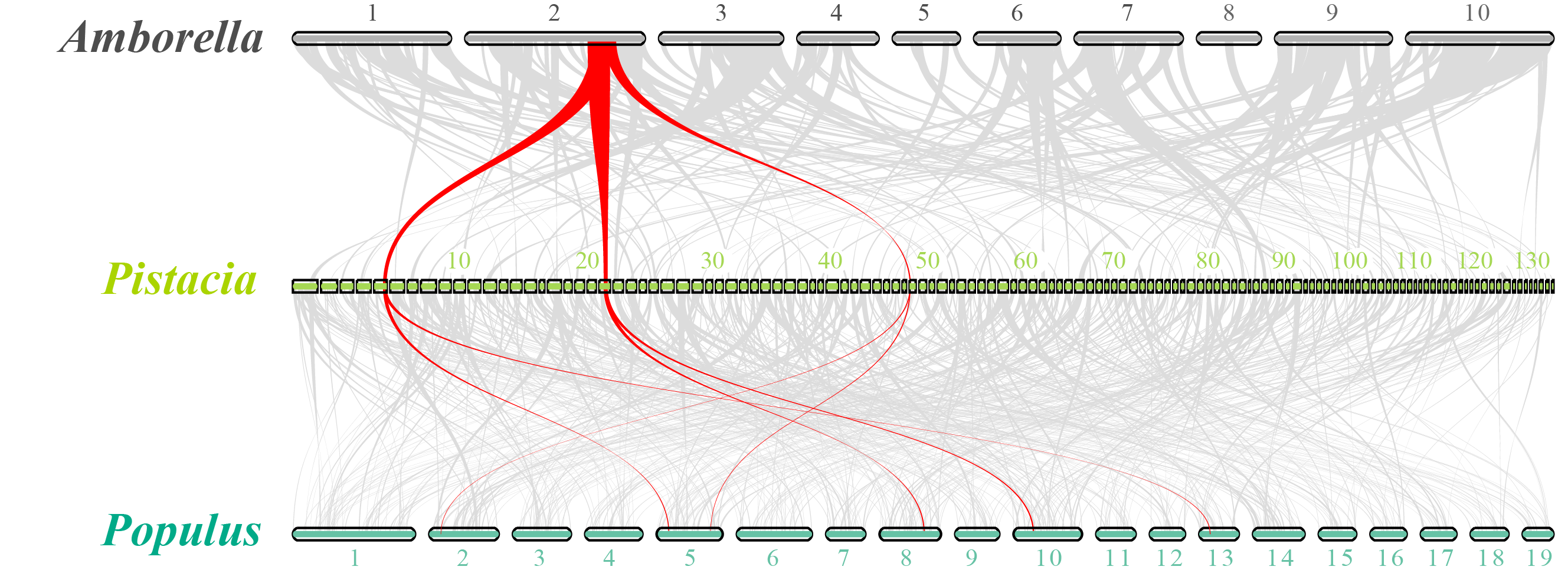


**Figure S3:** Macrosynteny analyses. No support for a lineage-specific genome duplication in pistachio is found in the synteny analysis, however, the gamma duplication occurring within eudicots, and a lineage specific genome duplication event in *Populus* is supported.

**
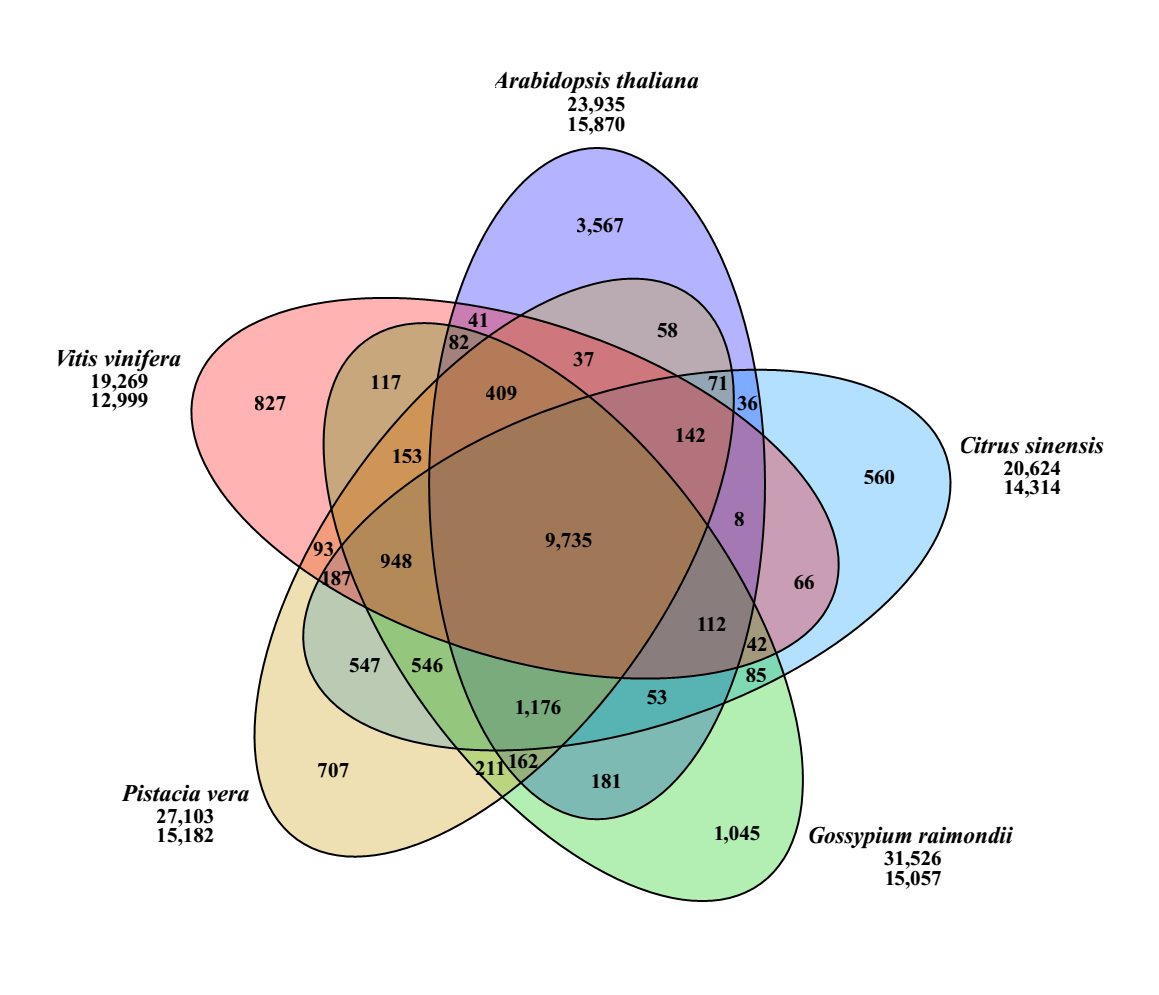
**

**Figure S4:** Unique and shared gene families among different plant species. Venn diagram shows the sharing of gene families among *P. vera*, *A. thaliana*, *C. sinensis*, *G. raimondii*, and *V. vinifera*. 9,735 gene families were found in all species, while 707 families, containing 1,381 genes, were specific to pistachio.

**
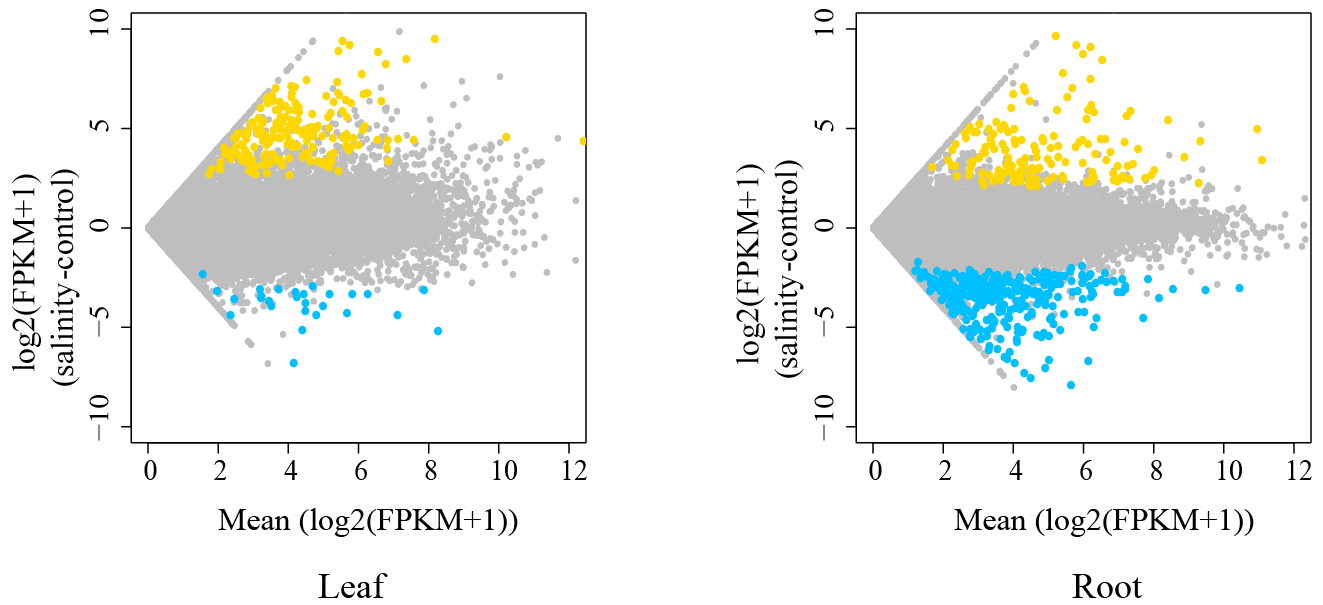
**

**Figure S5:** Comparison of gene expression in leaf (left) and root (right) tissue between salinity and control cultivated pistachio. Blue dots represent down-regulation and yellow dots represent up-regulated genes.


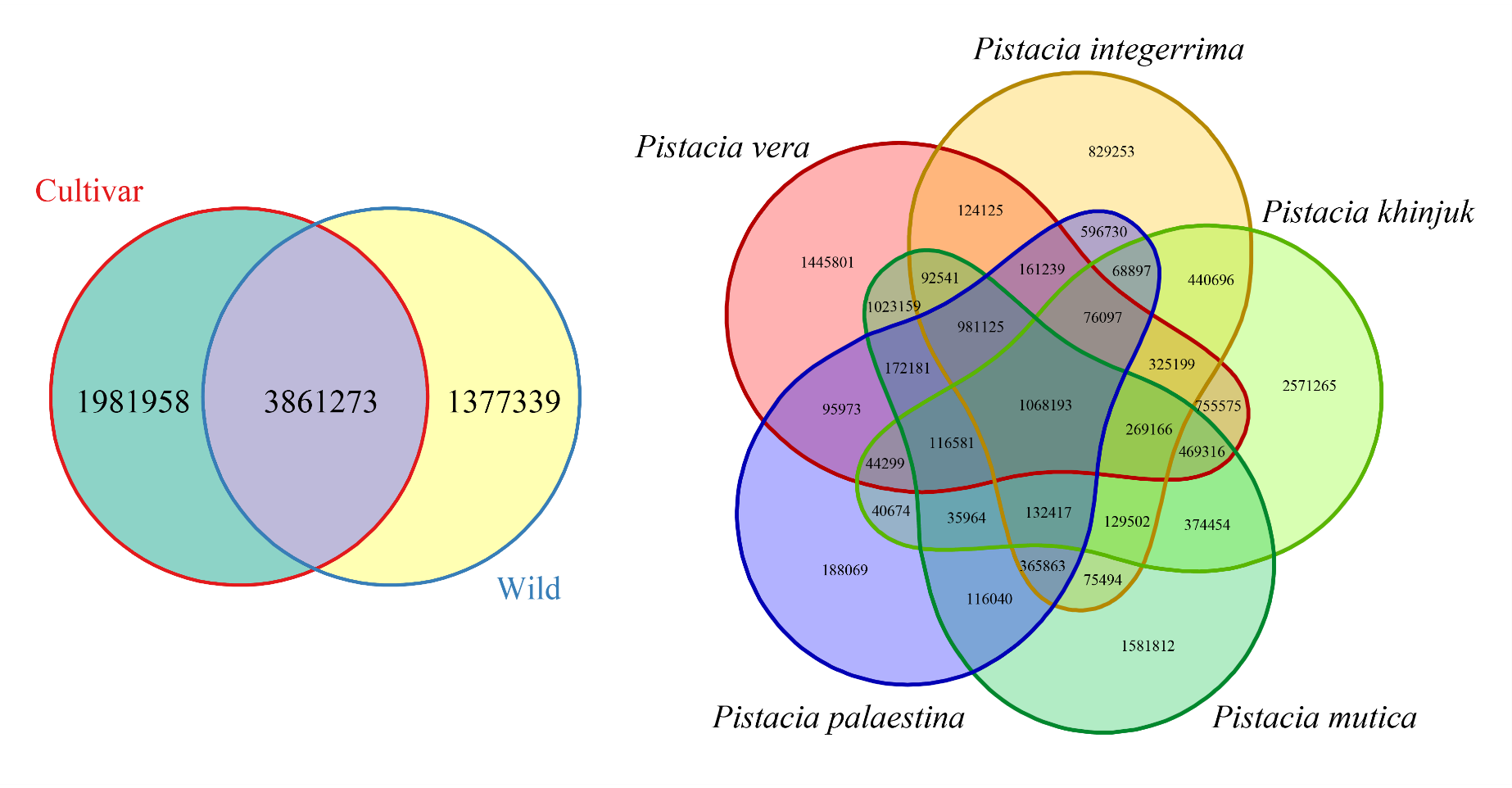


**Figure S6:** Venn diagram of unique and common single-nucleotide polymorphisms (SNPs) in different groups of pistachio. (left) Number of unique and shared SNPs in cultivated and wild pistachio. (right) Number of unique and shared SNPs in five different species, i.e., *P. vera, P. mutica, P. khinjuk, P. integerrima* and *P. palaestina*.

**
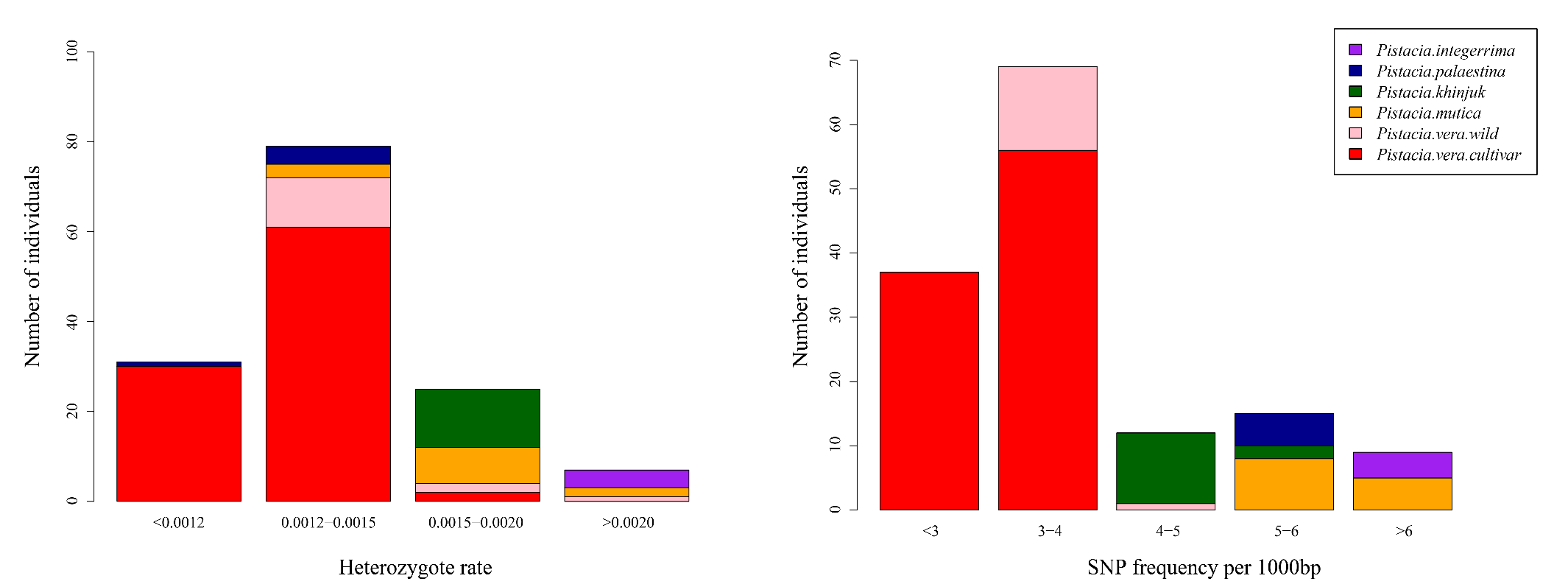
**

**Figure S7:** Heterozygosity at SNPs**.** (left) Heterozygote in five groups of pistachio and SNP frequency (right) for individuals of each species.

**
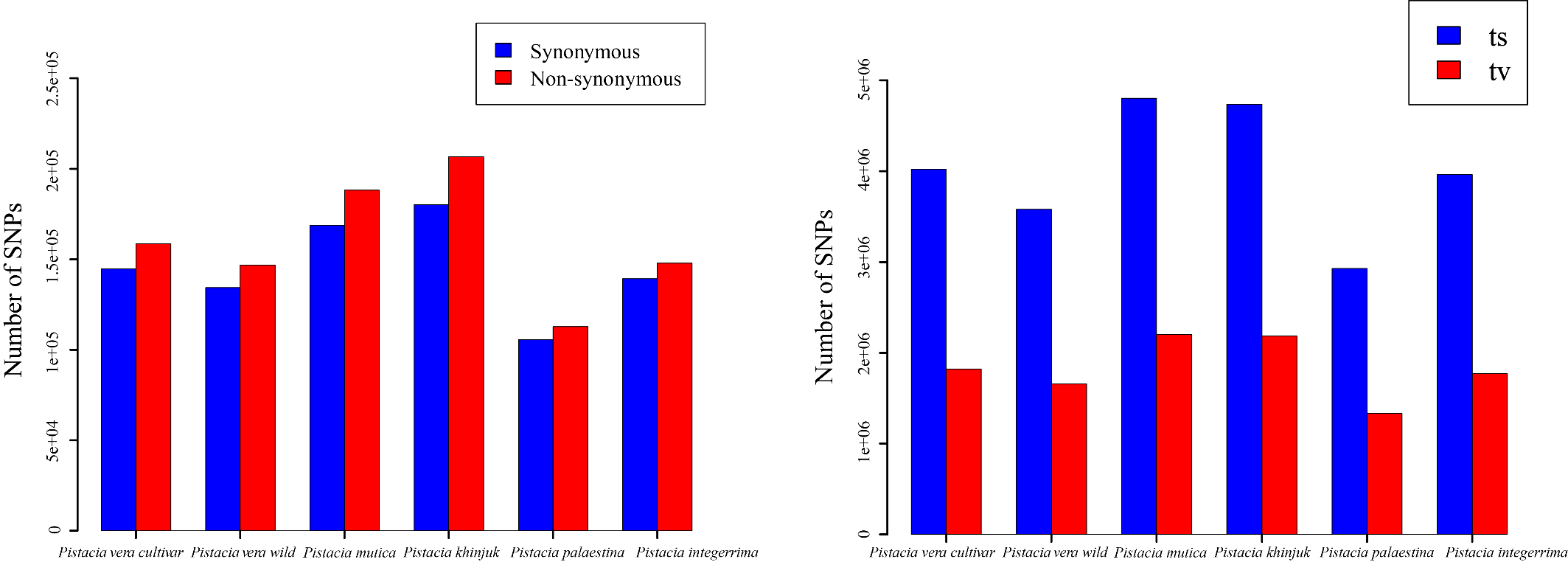
**

**Figure S8:** Numbers of synonymous and nonsynonymous SNPs (left) and numbers of transition (ts) and transversion (tv) (right) at the population level.

**
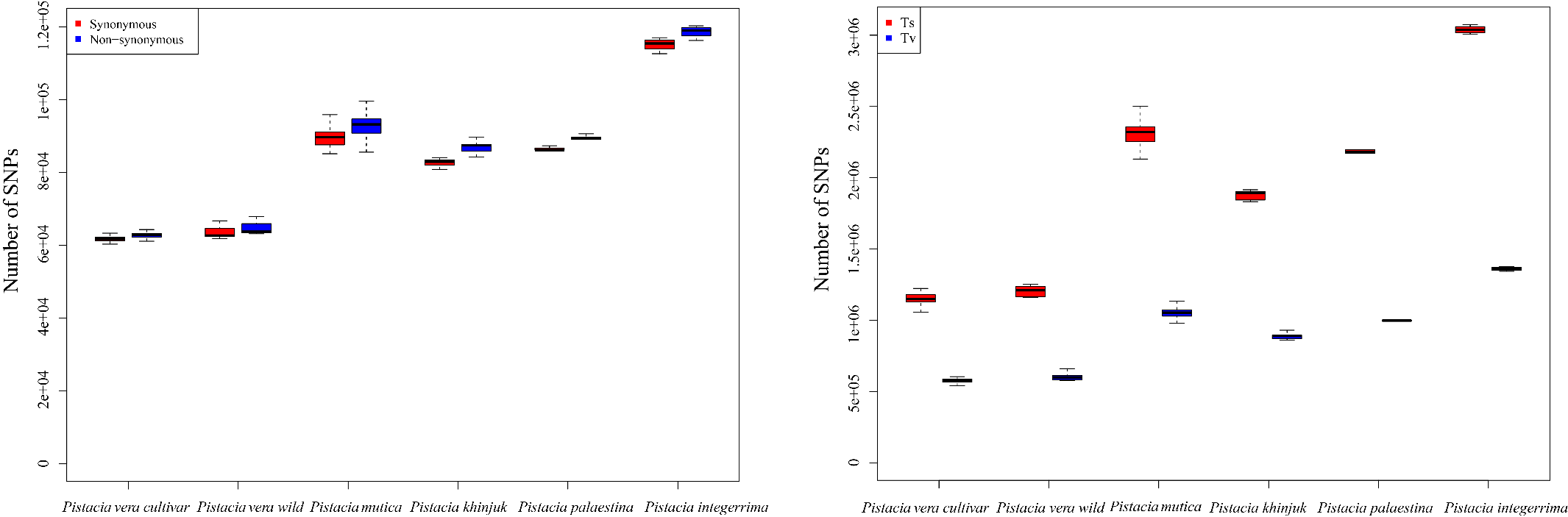
**

**Figure S9:** Numbers of synonymous and nonsynonymous SNPs (left) and transition (ts) and transversion (tv) (right) at the individual level.

**
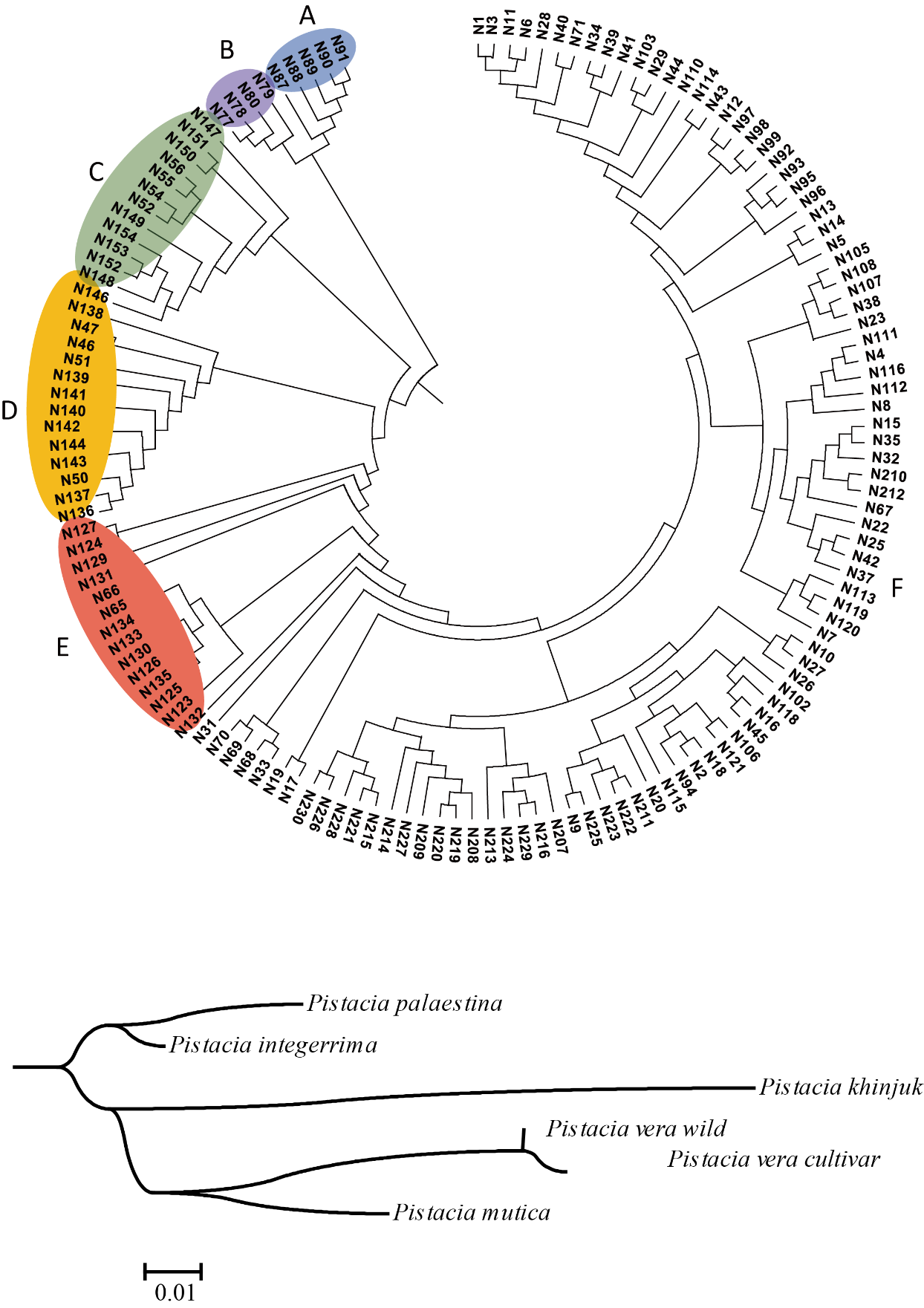
**

**Figure S10:** Phylogenetic analyses using the neighbor joining (up) and maximum likelihood (down) methods clearly separated the five different species, i.e. *P. palaestina* (A), *P. integerrima* (B), *P. khinjuk* (C), *P. mutica* (D) and *P. vera* (E & F).


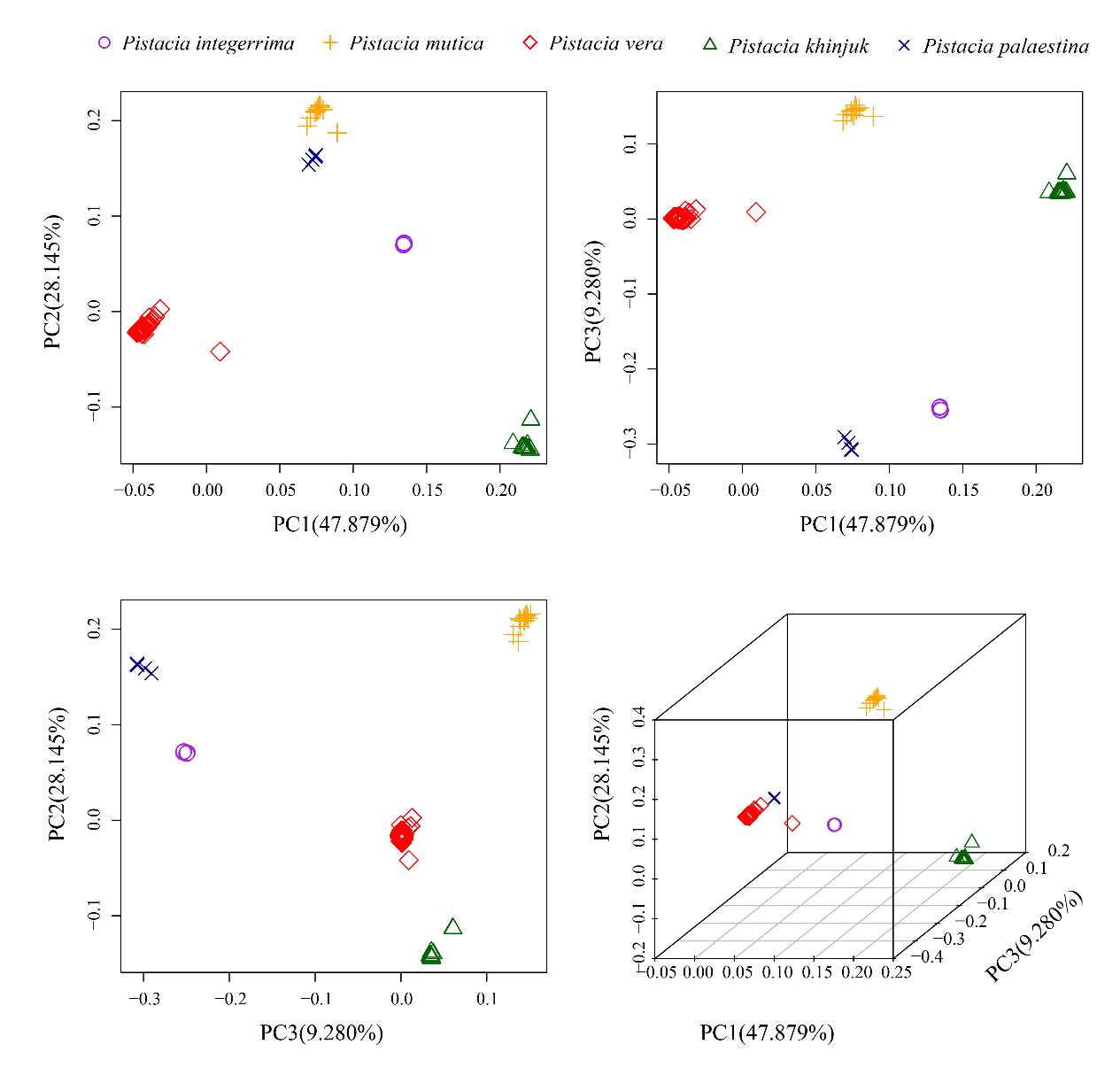


**Figure S11:** Principle component analysis clearly separated the five different *Pistacia* species.


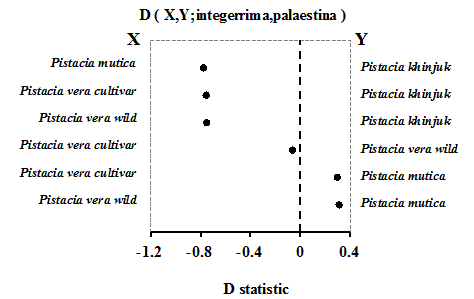


**Figure S12:** D (ABBA-BABA) test indicated introgression from P. khinjuk into P. integerrima.

**
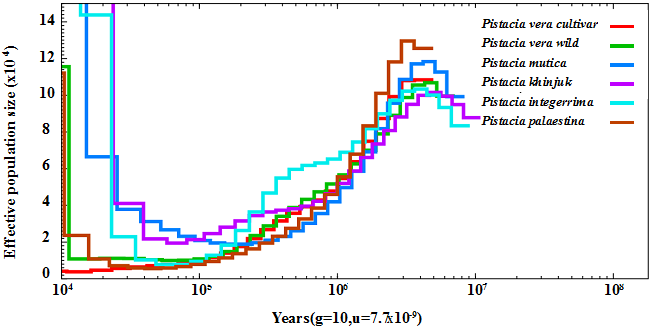

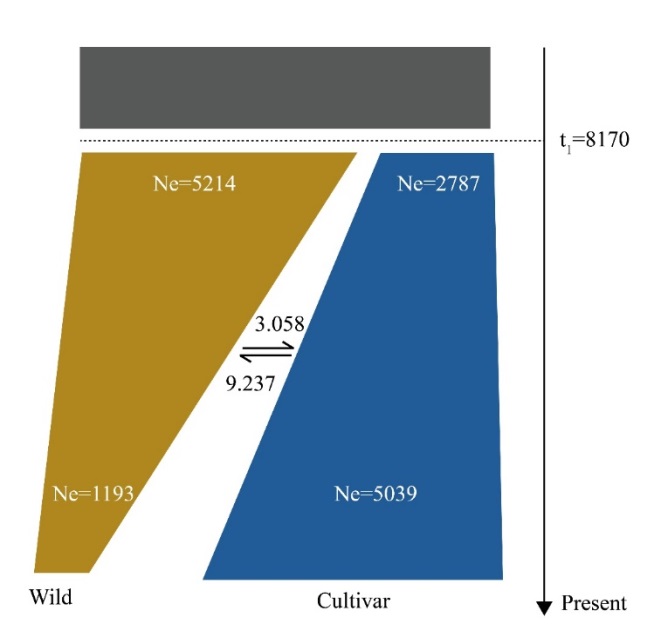
**

**Figure S13:** Dynamic change in effective population size inferred by PSMC (left). Analysis of the divergence time between wild and domestic pistachio using δaδi indicates that they separated ~8,000 year ago, which is similar to the archaeological record showing that pistachio seeds were a common food as early as 6,750 BC (right).

**
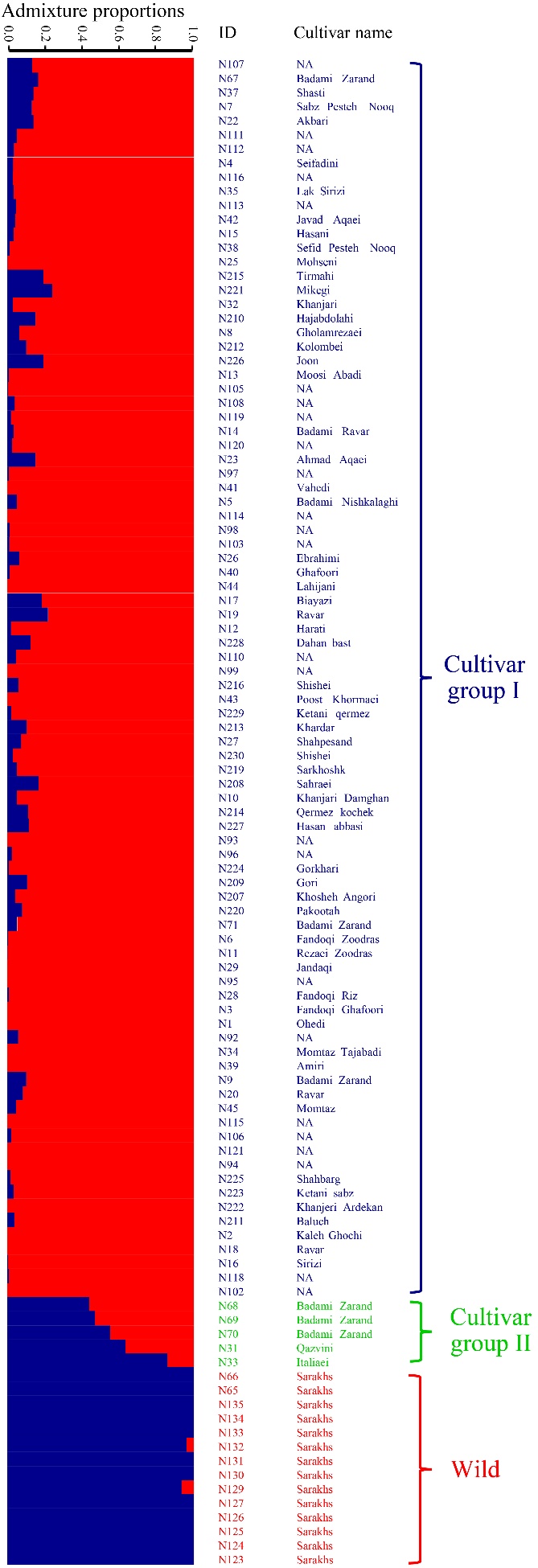
**

**Figure S14:** Admixture analysis of *P. vera* when 2 ancestral populations (K=2) is assumed.
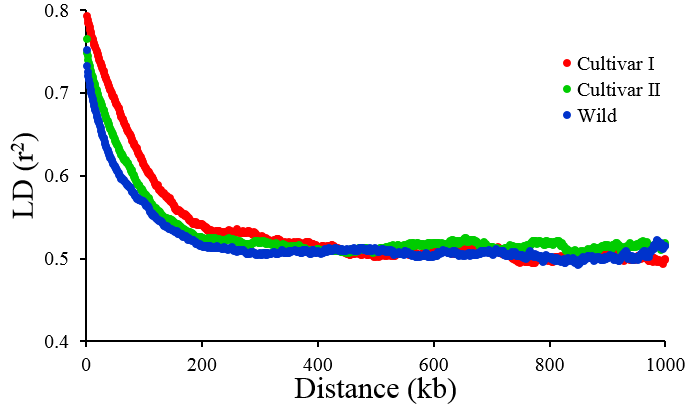


**Figure S15:** Decay of LD (linkage disequilibrium) of cultivar I, cultivar II and wild pistachio. The level of LD is highest within cultivar group I, while the rate of decay of LD is nearly the same in both cultivar group II and wild pistachio.

**
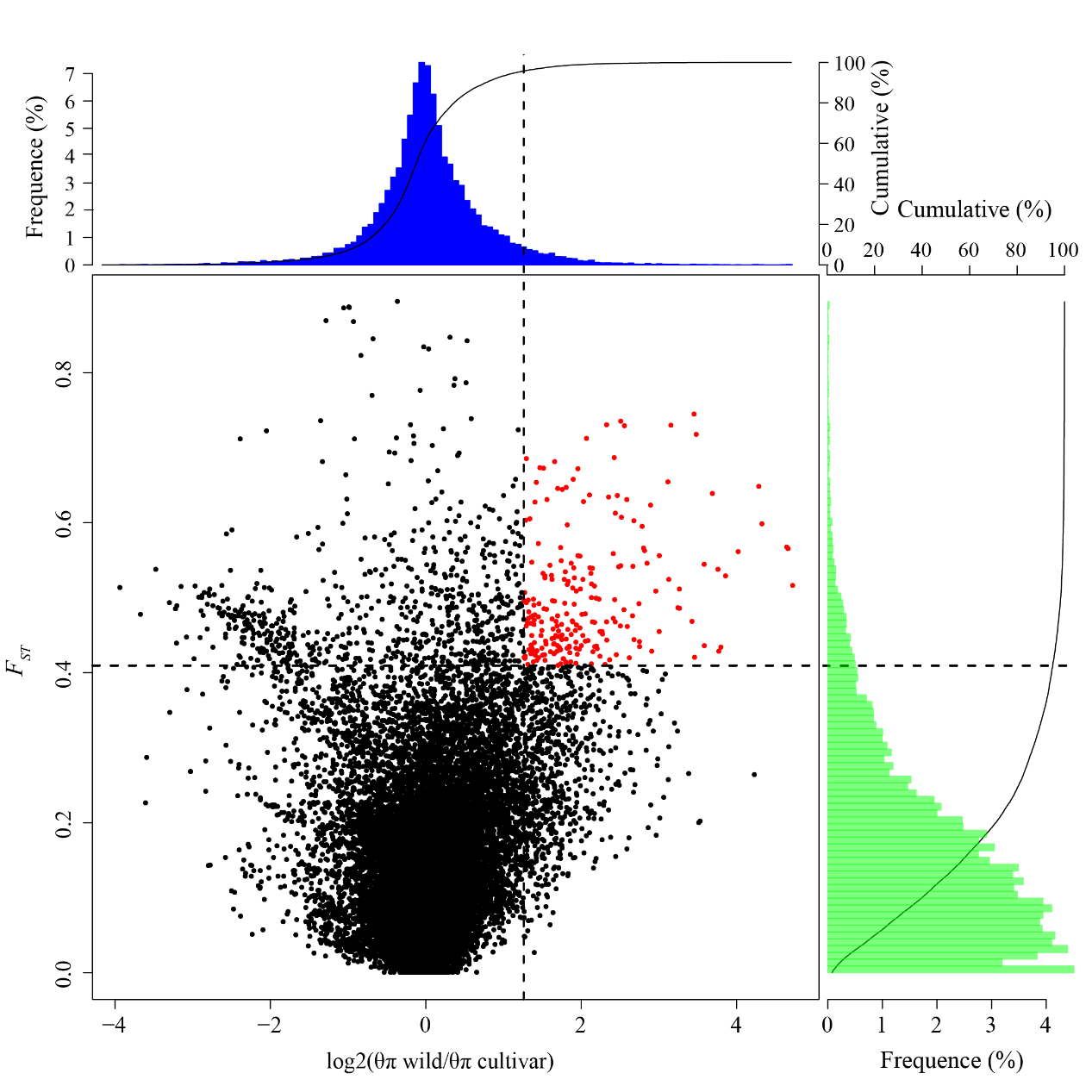
**

**Figure S16:** Distribution of population differentiation between domestic and wild pistachio and genetic diversity within cultivars. Dashed lines are 95% thresholds. Dots in red show both high population differentiation between populations and high diversity within cultivars, with both above the 95% threshold.


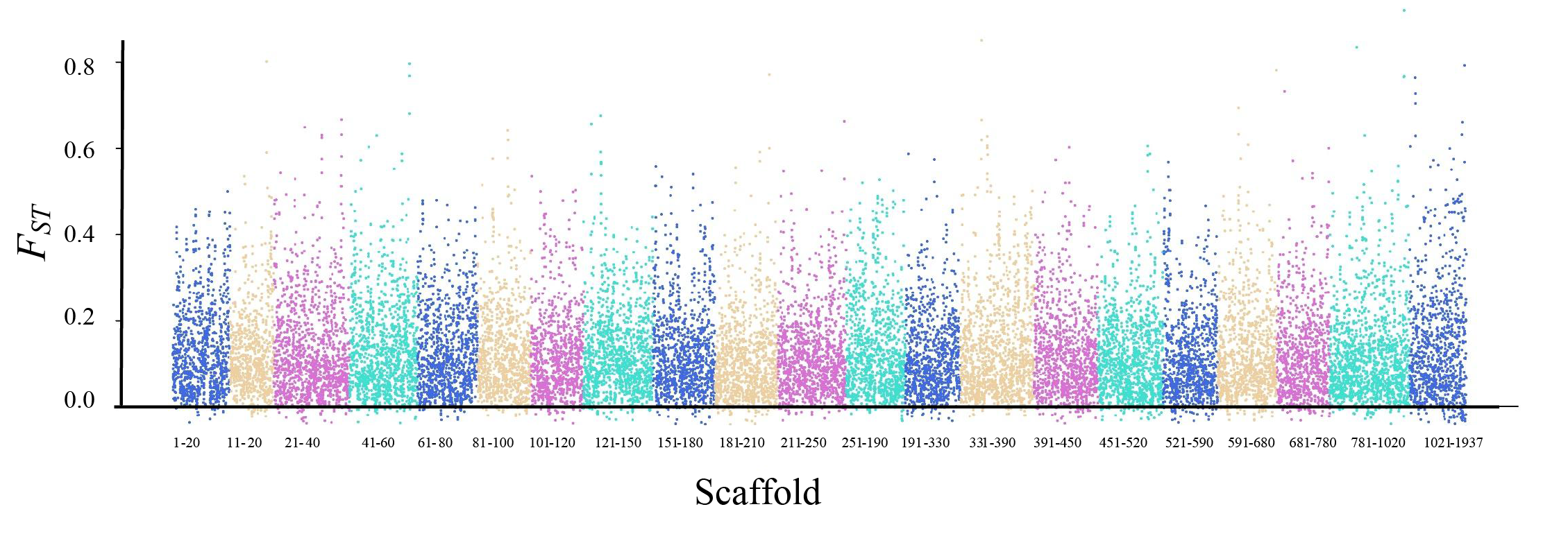


**Figure S17:** Level of differentiation (*F*_ST_ value) between cultivated and wild accessions of pistachio.

**
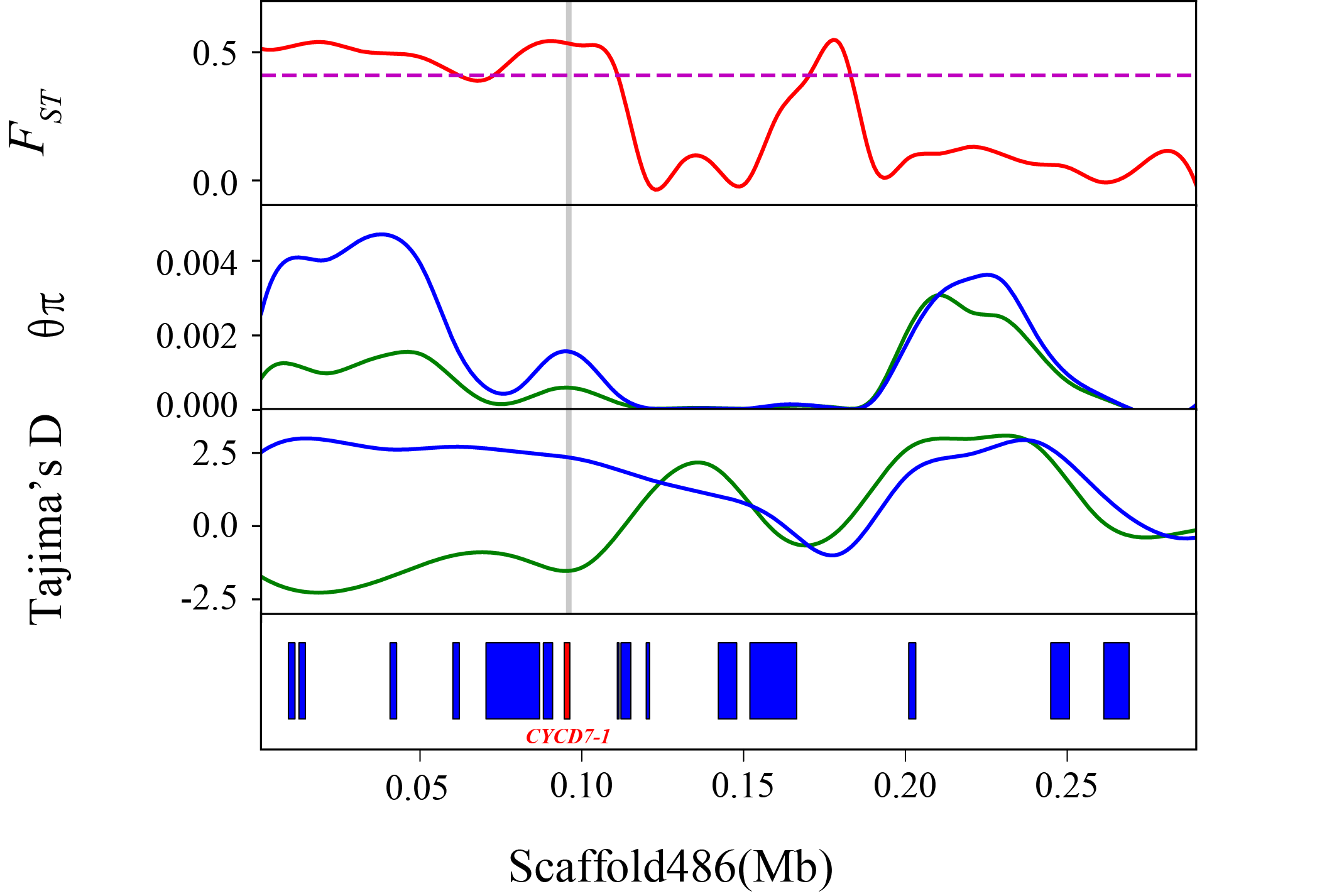
**

**Figure S18:** *F*_ST_*,* θπ and Tajima's D value for *CYCD7-1* between cultivated and wild pistachio. *CYCD7-1* evolved under artificial selection with a signature of a high level of population differentiation between wild and domestic cultivars. This gene encodes a D-type cyclin, which controls cell division and growth rate during seed development.


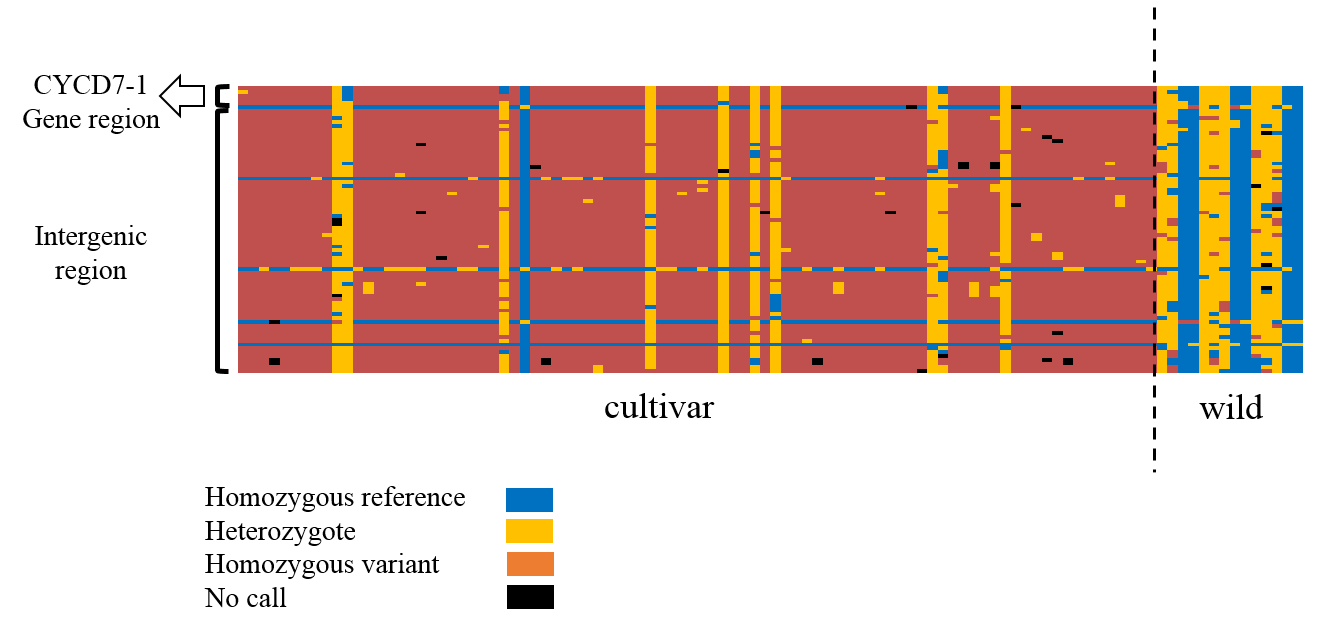


**Figure S19:** High level of population differentiation around *CYCD7-1* between wild pistachio and cultivar.


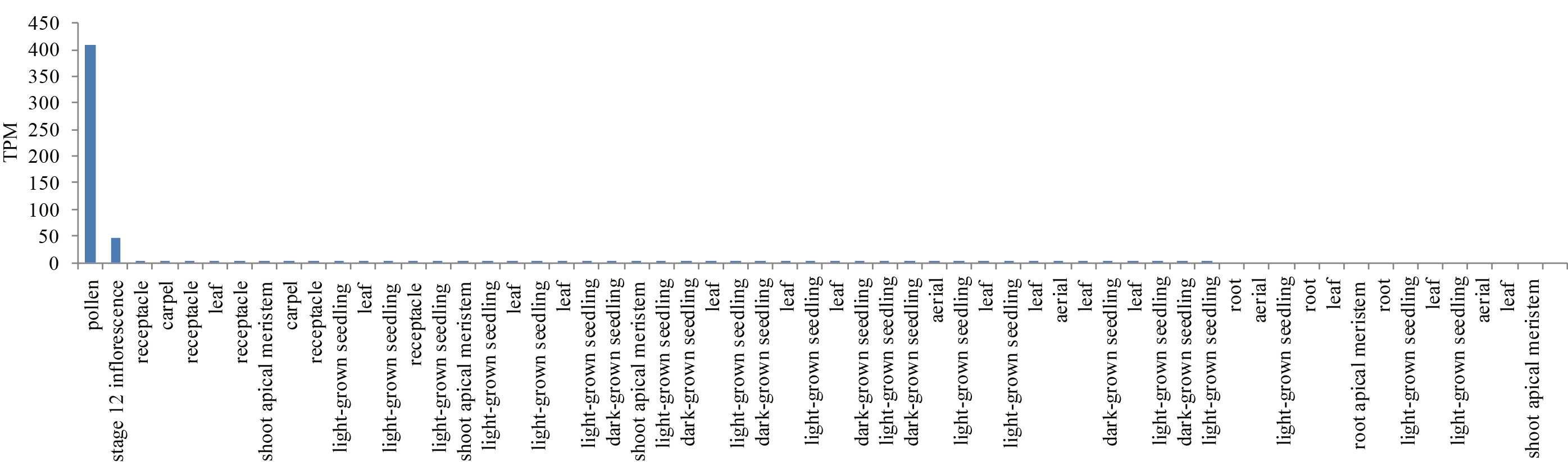


**Figure S20:** Expression pattern of gene *CYCD7-1* in different tissues.
